# Bone mineral density at age 7 years does not associate with adherence to vitamin D supplementation guidelines in infancy or vitamin D status in pregnancy and childhood: an Odense Child Cohort study

**DOI:** 10.1017/S0007114521000301

**Published:** 2021-11-28

**Authors:** Signe Monrad Nørgaard, Christine Dalgård, Malene Søborg Heidemann, Anders Jørgen Schou, Henrik Thybo Christesen

**Affiliations:** 1 Department of Clinical Research, Faculty of Health Sciences, University of Southern Denmark, Odense C, Denmark; 2 Hans Christian Andersen Children’s Hospital, Odense University Hospital, Odense C, Denmark; 3 IST – Clinical Pharmacology, Pharmacy and Environmental Medicine, University of Southern Denmark, Odense C, Denmark; 4 Steno Diabetes Center Odense, Odense University Hospital, Odense C, Denmark

**Keywords:** Bone mineral density, Vitamin D supplementation, 25-Hydroxyvitamin D, Children, Dual-energy X-ray absorptiometry

## Abstract

Vitamin D supplementation in infancy is recommended to prevent rickets. At the population level, its effects on bone mineralisation are largely unknown. We aimed to explore whether adherence to national vitamin D supplementation guidelines (10 µg/d up to the age of 2 years), supplementation at the ages of 5 and 7 years, and serum 25-hydroxyvitamin D (s-25(OH)D) at various time points associated with bone mineral density (BMD) at the age of 7 years in the Odense Child Cohort, Denmark (*n* 1194). High adherence was defined as supplementation with 10 µg of vitamin D 6–7 times per week during ≥80 % of the observation time. s-25(OH)D was analysed using LC-MS/MS. Total-body-less-head (TBLH) BMD was measured by dual-energy X-ray absorptiometry. At the median age of 18·1 months, 53·9 % (*n* 475/881) reported high adherence. The median s-25(OH)D was 64·7, 78·8, 46·0 and 71·8 nmol/l in early pregnancy, late pregnancy, cord blood and at 5 years, respectively. The mean TBLH BMD at the median age of 7·1 years was 0·613 (SD 0·049) g/cm^2^ (*z*-score +0·363 (SD 0·824)). In adjusted analyses, vitamin D supplementation up to 18 months, and at 5 and 7 years, was not associated with TBLH BMD. Similarly, no robust associations were found between TBLH BMD and s-25(OH)D at any time point. No associations were found for TBLH bone mineral concentration or bone area. In this population with relatively high s-25(OH)D concentrations, no consistent associations were found between adherence to vitamin D supplementation recommendations or vitamin D status in pregnancy or childhood, and bone mineralisation at the age of 7 years.

Vitamin D and Ca are important for ensuring optimal bone accretion in childhood and thereby maximising peak bone mass, which may prevent osteoporosis and bone fractures later in life^(1–4)^.

In fetal life, bone mineralisation is independent of vitamin D *per se*, but maternal calcitriol concentration increases during pregnancy, ensuring Ca supply to the fetus^(5)^. According to a recent global consensus report, a daily vitamin D supplementation of 15 µg is recommended for all pregnant women to prevent hypocalcaemia, dental enamel malformations and congenital rickets in offspring^(6)^.

Furthermore, all infants should, irrespective of diet, use vitamin D supplementation (10 µg per day from birth to the age of 12 months) to prevent nutritional rickets. In Denmark, a daily vitamin D supplementation of 10 µg has until January 2020 been recommended for pregnant women and all children up to the age of 24 months, regardless of vitamin D intake through diet and fortified formula milk, to support maximal bone growth^(7,8)^. Sun exposure is, through UV-B radiation, an important source for endogenous synthesis of vitamin D and thus a determinant for vitamin D status^(9)^. However, direct sun exposure is not recommended for neonates and infants and should be limited in older children to protect from skin cancer^(6,10)^.

Evidence is conflicting whether vitamin D supplementation in pregnancy improves growth or bone mass accretion in the offspring, although programming theories have been proposed^(11–18)^. In addition, randomised controlled trials (RCT) on the possible effect of vitamin D supplementation in healthy school-aged children on total body or total-body-less-head (TBLH) bone mineral content (BMC) and bone mineral density (BMD) report diverging results^(19–22)^, although the majority of the studies find no significant improvements at follow-up^(19,21,22)^.

In an RCT with vitamin D supplementation doses ranging from 10 to 40 µg given daily to children at the age of 1 month until 12 months, the total body BMD and BMC measured by dual-energy X-ray absorptiometry (DXA) scans at 1 and 3 years were similar between the groups^(23,24)^. However, an observational study on breastfed infants found an increase in bone mineralisation in children who used a daily supplementation of 10 µg of vitamin D compared with those who did not use supplementation^(25)^.

According to the Institute of Medicine, serum 25-hydroxyvitamin D (s-25(OH)D) concentrations above 50 nmol/l are sufficient to ensure optimal bone health in adults^(26)^. Vitamin D supplementation to adults with vitamin D sufficiency may therefore by redundant. However, less is known about this for children. In the Danish population-based Odense Child Cohort (OCC), where vitamin D status in pregnancy and childhood is relatively high, we have previously found that s-25(OH)D measured in early and late pregnancy and in cord blood was neither associated with offspring skull parameters, nor total length or height up to 3 years of age^(27,28)^.

Adherence to vitamin D supplementation recommendations may be far from optimal^(29,30)^, and the associations with bone mineralisation are largely unknown in children. Therefore, we examined the association between adherence to vitamin D supplementation recommendations for children up to at the age of 18 months and DXA TBLH BMD at the age of 7 years. Furthermore, we examined the association between DXA TBLH BMD and s-25(OH)D measured in early and late pregnancy, umbilical cord and at the age of 5 years. Finally, we examined the associations between vitamin D supplementation use at the ages of 5 and 7 years, and DXA TBLH BMD at the age of 7 years.

## Population and methods

The OCC design and protocol have been described previously^(31)^; briefly, all pregnant women (*n* 6707) living in Odense Municipality, Denmark, from 2010 through 2012 were invited to participate and 2876 women were enrolled.

Blood samples were collected from mothers in early and late pregnancy (before and after 20 weeks, respectively), in umbilical cord at birth, and from children at 5 years of age. Questionnaires on vitamin D supplementation, diet, physical activity, lifestyle, socio-demographic factors and diseases were collected twice during pregnancy and at 3 months, 18 months, 5 years and 7 years of age. The children were physically examined at the ages of 3 months, 18 months, 5 years and 7 years. To ensure that the children were of the same age at the time of the examinations, they were invited to participate at a date close to their birthday.

In the present study, OCC participants with either available data on vitamin D supplementation at the ages of 18 months, 5 years, or 7 years or s-25(OH)D at any age and available DXA scan at the age of 7 years were included. Exclusion criteria were multiple pregnancy, chronic diseases that may affect bone mineralisation and growth, for example, diabetes, leukaemia and neurodevelopmental diseases, and treatment with systemic corticosteroids for longer than 3 months in total. Children born pre-term were not excluded from the study unless being affected by other diseases; instead, we chose to adjust for gestational age at birth to account for potential effects on bone mineralisation.

### Assessment of adherence to recommendations on vitamin D supplementation

Intake of vitamin D supplementation was determined through a questionnaire at child age 18 months. Parents were asked to report whether their child used or had previously used vitamin D supplementation, and in that case further report duration and frequency. Children were defined as having high adherence if they used 10 µg of vitamin D supplementation 6–7 times per week during at least 80 % of the observation time. Non-adherence was defined as all intakes lower than that of the high-adherence group. The non-adherent group was further divided into intermediate adherence, defined as using vitamin D supplementation for longer than 6 months, but without fulfilling the criteria of the high-adherence group, and low adherence defined as using supplementation for 6 months or less.

### Assessment of vitamin D status

All blood samples were collected using BD Vacutainers serum clot activator tubes, reference 309 032. The blood samples were stored at −80°C until s-25(OH)D was analysed by the LC-MS/MS method as previously described^(32)^. The accuracy of the method was validated using Standard Reference Material 972 ‘Vitamin D in Human Serum’, the National Institute of Standards and Technology. s-25(OH)D_3_ was on average (target), for low-, mid- and high-level QC, 16·7 (16·9) nmol/l, 53·4 (54·7) nmol/l and 105·5 (107) nmol/l), respectively. The CV were 8·1, 5·5 and 6·6 %, respectively. Vitamin D_2_ was on average (target), for low-, mid- and high-level QC, 16·2 (15·3) nmol/l, 51·7 (49·1) nmol/l and 103·9 (97·1) nmol/l. The CV were 6·9, 5·1 and 6·1 %, respectively. C3-epimers were included in the s-25(OH)D measurements. In a tested subsample, C3-epimer concentrations in pregnancy and cord blood were between 1·1 and 3·0 nmol/l, that is, 0·5–2·3 % of total s-25(OH)D concentration^(33)^.

### Assessment of bone mineral density

The primary outcome was TBLH BMD at 7 years of age, obtained by whole-body DXA scans using a Prodigy DXA scanner with enCORE software version 17 (GE Healthcare, General Electric Company). The children were positioned on the back, wearing only underpants, while the scan was performed in one movement from head to toes with an estimated entrance skin dose of 0·4 µGy. The biomedical laboratory scientists who performed the scans ensured that the children were positioned correctly and laying still during the scan; if this was not the case, the scan was repeated. The enCORE software automatically adjusted placement of landmarks. All scans were assesed by a biomedical laboratory assistant who checked the positioning and the quality and evaluated if it was nescessary for a paediatrician to also assess the scan. The precision and reproducibility of the DXA scanner were tested by daily scans of a phantom and additional controls once per month following the guideline from the manufacturer. The CV was between 0·2 and 0·4 %. As the DXA scans were performed as part of the physical examination close to the children’s 7-year birthdays, they were performed year round.

### Assessment of covariates

At child age 3 months, skintone was determined at the buttock through examination using the Fitzpatrick scale^(34)^. At the age of 7 years, barefooted height was measured in centimetres with 1-decimal precision using a Seca 213 portable stadiometer (SECA International). Child weight was measured wearing minimal clothing in kg with 1-decimal precision using Seca 861 digital scales. BMI was calculated by weight in kg divided by height in metres squared. Pubertal stage was determined by physical examination (inspection and palpation) of breast tissue and testicle volume using an orchidometer according to the Tanner scale^(35,36)^. All physical examinations were conducted by trained biomedical laboratory scientists.

Also at child age 7 years, parents were asked to indicate by categories rounded to intergers (1–2 dl, 3–4 dl, 5–6 dl and >6 dl) the amount of dairy products (milk and yogurt) the child consumed daily, using a survey question from the Danish National Birth Cohort.^(37)^ The children were grouped into those consuming at least 3 dl and thus adhered to the recommendations of the Danish Health Authorities (≥2·5 dl) and those consuming less. Furthermore, parents were asked to indicate how many days of a normal month the child would eat meat for dinner. Finally, the parents were asked to evaluate how active the child was compared with other children of the same age and sex by selecting one of the following options: ‘far less active’, ‘a little less active’, ‘equally active’, ‘a little more active’ or ‘far more active’. These options were reduced to ‘less active’, ‘equally active’ and ‘more active’. The question was inspired from the questions used in Sallis *et al.*^(38)^

Information on the following parameters was validated using medical records: mother’s age and parity after delivery, smoking in pregnancy, gestational age at birth, sex, and child body length and weight at birth.

### Statistical analyses

Differences between the high-adherent and non-adherent groups, participants and non-participants, and participants with complete and incomplete covariate data were tested using the two-sample Wilcoxon rank sum (Mann–Whitney) test on non-normally distributed variables, Pearson’s *χ*^2^ test on categorical variables and the two-sample *t* test with equal variances on normally distributed variables.

We used multiple linear and logistic regressions to examine the association between adherence to vitamin D supplementation recommendations and BMD at the age of 7 years. In our primary analyses, we used adherence at the age of 18 months as the exposure and TBLH BMD at the age of 7 years as the outcome. As the BMD *z*-score differed from the reference population, we instead used TBLH BMD adjusted for height and child sex as mandatory variables in the basic model 1. Also, the mandatory variable skin tone was considered for this model as an indicator of ethnicity but was instead included in model 3 due to missing values. Model 2 further adjusted for gestational age, parity and child BMI. Model 3 adjusted for all the above as well as physical activity, skin tone and daily dairy product consumption, as information on these covariates were missing for some of the participants.

The covariates included in the models were chosen *a priori* based on a review of the literature and their association with either the exposure or outcome. Covariates associated with exposure were gestational age and parity. Alcohol consumption in pregnancy was not included as very few participants consumed alcohol more than once. Smoking, maternal education level and season of medical examinations were not included as they were neither associated with the exposure nor outcome. Covariates univariately associated with the outcome were child height, child weight (BMI), daily intake of dairy products and amount of physical activity compared with peers as estimated by the parents (all measured at the age of 7 years).

We performed the analyses defined *a priori* stratified by child sex and tested for possible effect modification because sex differences in bone mineralisation have been reported already in childhood^(39,40)^. Furthermore, we examined the association between the use of vitamin D supplementation in pregnancy and at the age of 5 years and s-25(OH)D in early pregnancy, late pregnancy and at the age of 5 years, respectively, by applying multiple linear regression adjusting for the same covariates as in model 3. Finally, we performed multiple logistic regressions with BMD categorised as low (BMD ≤ 10th percentile) or normal (BMD > 10th percentile) using the same adjustments as in the linear regressions. The same approach was used when analysing the *a priori* defined secondary exposures, substituting adherence at the age of 18 months with s-25(OH)D concentrations in early and late pregnancy, in umbilical cord, and at the age of 5 years as well as the vitamin D supplementation use at the ages of 5 and 7 years.

When investigating relevant interactions in the models, interactions were found between skin tone and dairy product consumption for vitamin D adherence at the age of 18 months, and between physical activity and BMI for s-25(OH)D at the age of 5 years. Adding these interactions to the models did, however, not change any results (results not shown).

Model assumptions were evaluated for the final model 3 by checking normality and homoscedasticity of residuals, multi-collinearity, linearity and model specifications. As none of the participants were siblings, all observations were independent. Child BMI took the place of weight in all regression models, after which no assumptions were violated. Sensitivity analyses were carried out using weight instead of BMI. This did not change any of the results (results not shown).

Two-sided *P* values < 0·05 were defined as statistically significant. With a sample size of 881 participants, a power of 80 % and the TBLH BMD standard deviation of 0·05, the minimal detectable difference was 0·09 g/cm^2^ between those who adhered to the guidelines and those who did not. Lastly, a backwards multiple linear regression analysis was performed to identify predictors for TBLH BMD. Data were analysed using Stata/IC 16 (StataCorp, LLC).

### Ethical considerations

The present study was conducted according to the guidelines laid down in the Declaration of Helsinki and all procedures involving human subjects were approved by the Regional Scientific Ethical Committee for Southern Denmark (no. S-20090130) and the Institutional Review Board (no. 18/47377). Written informed consent was obtained from all parents of subjects. Parents were informed about the risks of radiation before DXA scans were conducted. Participation was voluntary for all children and consent could be withdrawn at any time. Data were stored in Odense Patient Data Exploratory Network (OPEN) and in Odense Municipality’s Data Warehouse.

## Results

**Fig. 1.**
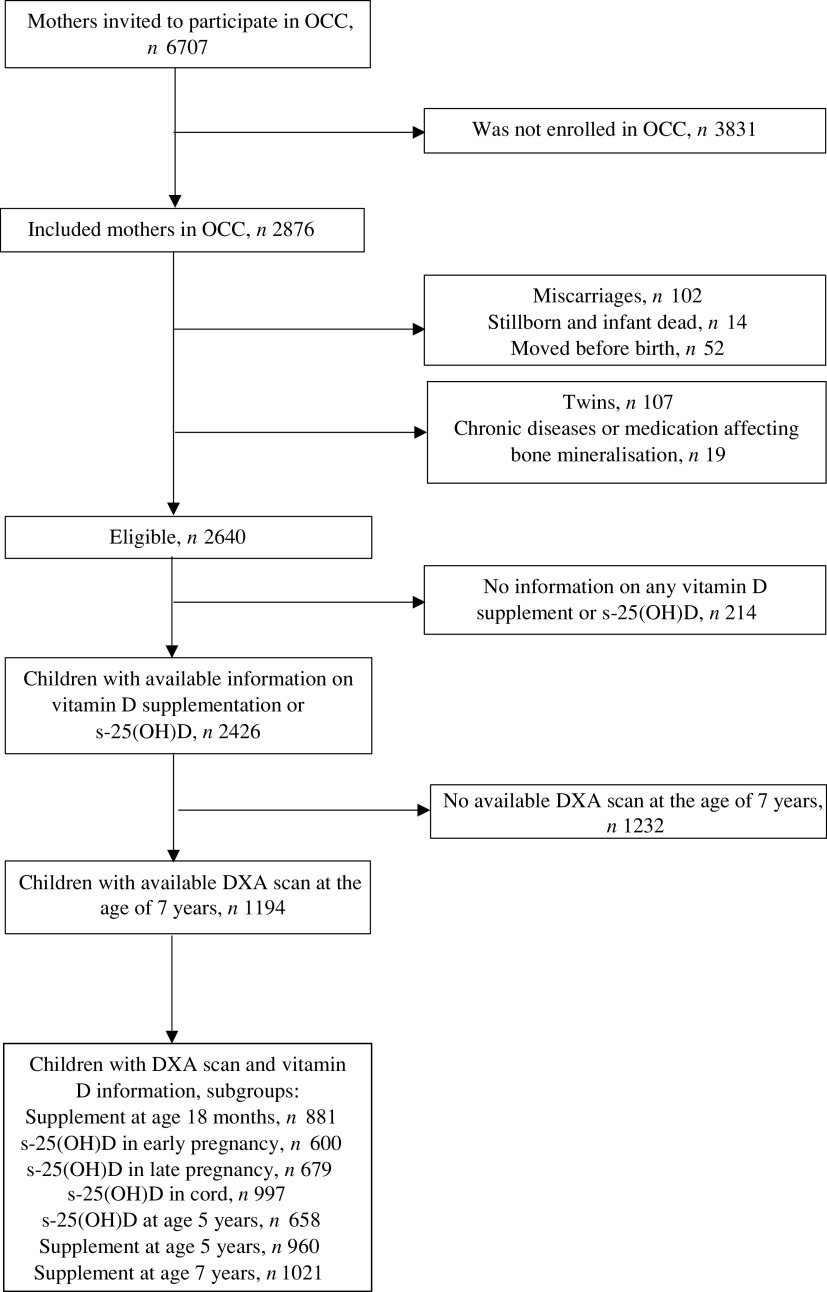
Participant inclusion flow chart. OCC, Odense Child Cohort; s-25(OH)D, serum 25-hydroxyvitamin D; DXA, dual-energy X-ray absorptiometry.

Of the 2876 participants in OCC, 2640 subjects were eligible for participation in the present study. Of these, 1194 participants (45·2 %) were finally included by having available DXA scans at the age of 7 years and data on at least one of the following parameters: vitamin D supplementation up to the ages of 18 months, 5 years or 7 years; or s-25(OH)D in early pregnancy, late pregnancy, umbilical cord or at the age of 5 years (Fig. 1). The characteristics of participants are presented in Table 1. The median age when answering the 18- month questionnaire was 18·1 (interquartile range (IQR) 1·3) months. The median age at the time of the DXA scan was 7·1 (IQR 0·1) years. All participants were pre-pubertal (Tanner stage 1) and primarily of Western origin. No participants were diagnosed with rickets at any time point.

**Table 1.** Descriptive characteristics of participants in the 18-month adherent and non-adherent groups* (Mean values and standard deviations; numbers and percentages; median values and interquartile ranges (IQR))

	Total				High adherence				Non-adherence				*P*†
	*n*	%	Median	IQR	*n*	%	Median	IQR	*n*	%	Median	IQR	
Number of participants	881				475	53·9			406	46·1			
Mother’s age at delivery (years), *n* 881			31·0	6·0			31·0	6·0			30·0	6·0	0·36
Mother’s parity, *n* 881													0·001
Unipara‡	464	52·7			307	64·6			157	38·7			
Maternal education, *n* 876													
Lower	232	26·5			121	25·6			111	27·5			0·76
Intermediate	437	49·9			241	51·0			196	48·6			
Higher	207	23·6			111	23·5			96	23·8			
Smoking in pregnancy, *n* 876													
Yes	38	4·3			18	3·8			20	5·0			0·40
Alcohol in pregnancy, *n* 594													0·01
Yes	74	12·5			30	9·3			44	16·3			
Vitamin D suppl. in pregnancy§, *n* 474													0·05
Yes	406	85·6			231	88·5			175	82·2			
Age at DXA scan (years), *n* 881			7·1	0·1			7·1	0·1			7·1	0·1	0·41
Sex, *n* 881													0·35
Male	458	52·0			240	50·5			188	46·3			
Skin tone, Fitzpatrick scale, *n* 829													0·53
Fitzpatrick scale 1–2	449	54·2			252	55·1			197	53·0			
Season at 18-month questionnaire, *n* 881													0·45
November–April	383	43·5			212	44·6			171	42·1			
TBLH BMD (g/cm^2^), *n* 881													0·57
Mean	0·613				0·613				0·614				
sd	0·049				0·050				0·048				
TBLH BMC (g), *n* 881			624·75	134·7			619·65	133·5			623·22	137·1	0·57
TBLH BA (cm^2^), *n* 881	1028	93·80			1025·21	91·48			1032·07	96·42			0·28
Mean	0·613				0·613				0·614				
sd	0·049				0·050				0·048				
TBLH *z*-score, *n* 881													0·48
Mean	0·364				0·346				0·385				
sd	0·816				0·816				0·833				
Height (cm), *n* 881													0·61
Mean	125·6				125·6				125·7				
sd	5·18				5·13				5·23				
Weight (kg), *n* 881			24·2	4·7			24·1	4·6			24·0	4·8	0·81
Child BMI (kg/m^2^), *n* 881			15·3	1·9			15·3	1·8			15·4	2·0	0·98
Physical activity, *n* 791													0·85
Less active	64	8·1			35	8·1			29	8·1			
As active	533	67·4			287	66·6			246	68·3			
More active	194	24·5			109	24·5			85	23·6			
Meat intake (d/month) (7 years), *n* 765			22·0	10·0			23·0	10·0			21·0	11·0	0·57
Daily dairy product consumption (7 years), *n* 781													
≤3 dl/d	343	43·9			189	44·1			154	43·8			0·93
Gestation age at birth (weeks), *n* 879			40·1	1·7			40·1	1·9			40·3	1·9	0·03
Body weight at birth (g), *n* 879			3550	662·0			3515	640·0			3580	690·0	0·20
Body length at birth (cm), *n* 873			52·0	2·0			52·0	3·0			52·0	2·0	0·35
Exclusive breast-feeding (weeks), *n* 874			13·0	16·0			13·0	16·0			12·0	16·0	0·67
Vitamin D suppl. at the age of 7 years§, *n* 783													0·006
≤1 time per week	411	52·5			204	47·6			207	58·5			
≥2 times per week	335	42·8			200	46·6			135	38·1			
Unsure of frequency	37	4·73			25	5·83			12	3·39			
Vitamin D suppl. at the age of 5 years§, *n* 762													0·001
≤1 time per week	378	49·6			179	43·1			199	57·4			
≥2 times per week	384	50·4			236	56·9			148	42·7			
Holiday weeks||, *n* 881			0	0			0	0			0	0	0·97

DXA, dual-energy X-ray absorptiometry; Suppl., supplementation; TBLH BA, total-body-less-head bone area; TBLH BMC, total-body-less-head bone mineral content; TBLH BMD, total-body-less-head bone mineral density.

* High adherence was defined as consuming 10 µg of vitamin D supplementation for 6–7 times per week during at least 80 % of the observation time, and non-adherent as otherwise.

† Differences between the high-adherent and the non-adherence groups were tested using the two-sample Wilcoxon rank sum (Mann–Whitney) test on non-normally distributed variables, Pearson’s χ^2^ test on categorical variables and the two-sample *t* test with equal variances on normally distributed variables.

‡ Mothers carrying their first child during the studied pregnancy.

§ Data on vitamin D supplementation in pregnancy and at the ages of 5 and 7 years could not be specified in more detail with regard to doses.

|| Weeks during the winter half-year spend by the child (aged 3–7 years) in countries with average monthly UV index high enough that the skin produces vitamin D.

### Adherence to vitamin D supplementation recommendation up to the age of 18 months

**Fig. 2.**
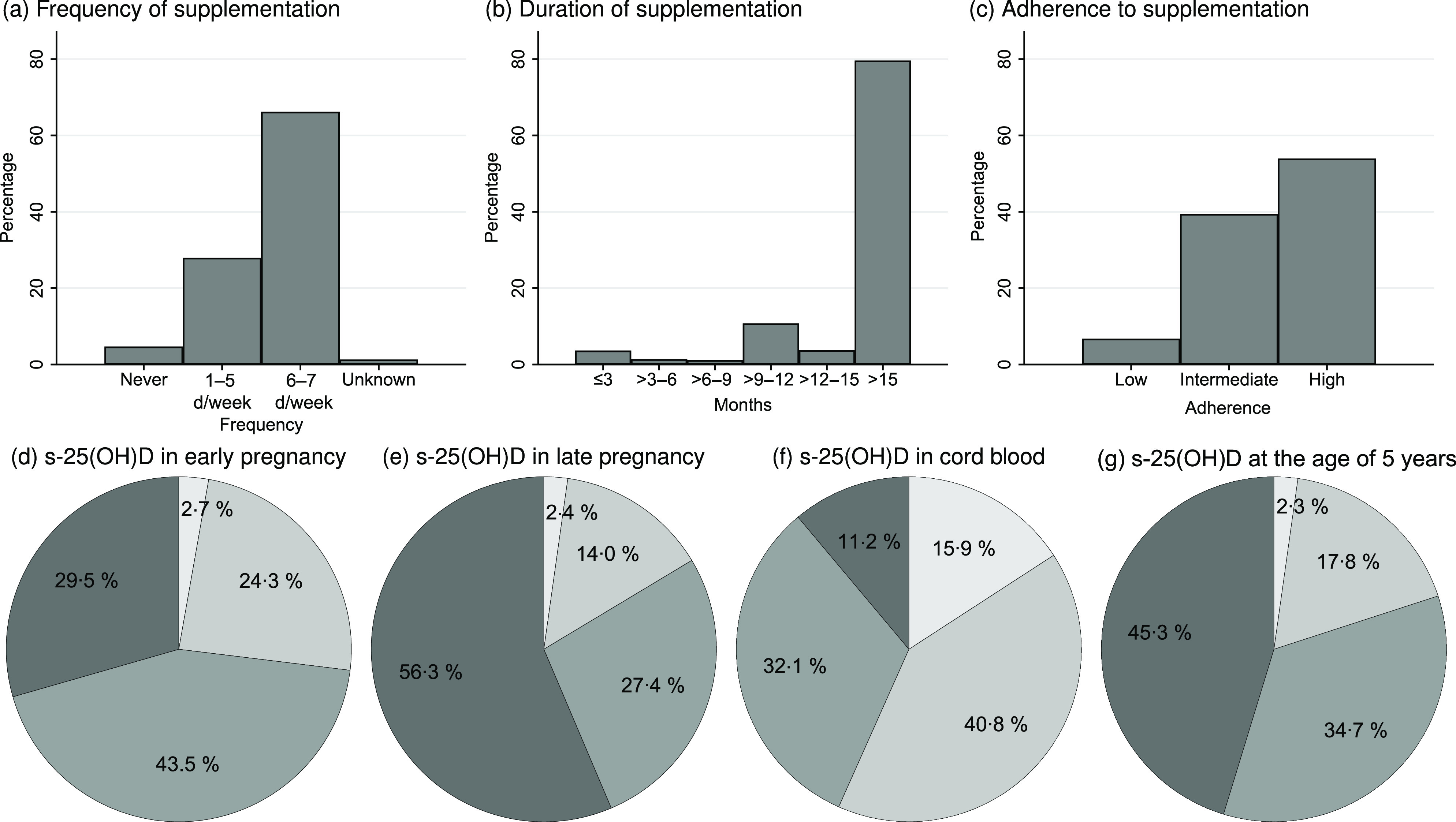
(a) Frequency and (b) duration of vitamin D supplementation, and (c) adherence to vitamin D supplementation recommendations for children up to the age of 18 months; serum 25-hydroxyvitamin D (s-25(OH)D) in (d) early pregnancy (before week 20 of pregnancy), (e) late pregnancy (after week 20 in pregnancy), (f) umbilical cord blood and at (g) the age of 5 years. 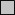, <25 mmol/l; 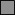, 25–50 mmol/l; 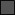, ≥50–75 mmol/l; 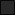, >75 mmol/l.

In total, 881 participants had DXA scans and information on vitamin D supplementation use up to the median age of 18·1 (IQR 1·3) months. Of these, 583 (66·2 %) participants used 10 µg of vitamin D supplementation 6–7 d per week (Fig. 2(a)), and 626 (71·1 %) participants still used vitamin D supplementation at the age of 18 months (Fig. 2(b)). High adherence to the national vitamin D supplementation recommendations was seen in 475/881 (53·9 %) of the participants (Fig. 2(c)). Only fifty-nine (6·7 %) were in the low-adherence group. Predictors for low or intermediate adherence included higher parity, higher gestational age and alcohol consumption in pregnancy (Table 1). High adherence to vitamin D supplementation recommendation in infancy was positively associated with vitamin D supplementation use at 5 and 7 years.

### Serum 25-hydroxyvitamin D concentrations

The s-25(OH)D concentrations at the different time points are depicted in Fig. 2(d)–(g). The median s-25(OH)D concentrations were: early pregnancy 64·7 (IQR 28·4) nmol/l (*n* 600), late pregnancy 78·8 (IQR 35·9) nmol/l (*n* 679), umbilical cord 46·0 (IQR 29·7) nmol/l (*n* 997) and 5 years of age 71·8 (IQR 32·8) nmol/l (*n* 658).

### Dual-energy X-ray absorptiometry scan results

Among our participants (*n* 1194), the mean TBLH BMD was 0·613 (SD 0·049) g/cm^2^ with a *z*-score of +0·363 (SD 0·824). The median TBLH BMC was 624·748 (IQR 135·255) g. The mean TBLH bone area (BA) was 1029·308 (SD 94·130) cm^2^.

### Associations between vitamin D supplementation and serum 25-hydroxyvitamin D

In pregnancy and at 5 years, a strong association between vitamin D supplementation and s-25(OH)D was observed (early pregnancy; *β*-coefficient = 9·37 (95 % CI 2·16, 16·59), *P* = 0·01 late pregnancy; *β*-coefficient = 8·90 (95 % CI 1·32, 16·48), *P* = 0·02 and 5 years; *β*-coefficient 3·79 (95 % CI 2·88, 4·70), *P* < 0·001).

### The association between vitamin D supplementation adherence in infancy and dual-energy X-ray absorptiometry scans

No associations were found between adherence to vitamin D supplementation in infancy and TBLH BMD at the age of 7 years (Table 2). Likewise, adherence to vitamin D supplementation did not associate with TBLH BMD *z*-score, BA or BMC (Table 3). Stratification by sex did not change any of the results (results not shown).

**Table 2.** Association between adherence to vitamin D supplementation at the age of 18 months and total-body-less-head bone mineral density (TBLH BMD; g/cm^2^) at the age of 7 years*† (*β*-Coefficients, odd ratios and 95 % confidence intervals)

TBLH BMD, continuous	Model‡	*β*	95 % CI	*P*
High adherence *v*. non-adherence	Model 1, *n* 881	0·001	−0·004, 0·006	0·69
	Model 2, *n* 881	−0·000	−0·005, 0·005	0·92
	Model 3, *n* 737	−0·001	−0·006, 0·004	0·68
High adherence *v*. low adherence	Model 1, *n* 534	0·004	−0·007, 0·015	0·50
	Model 2, *n* 534	−0·001	−0·012, 0·009	0·82
	Model 3, *n* 454	0·003	−0·009, 0·015	0·65
TBLH BMD, ≤10th *v*. >10th percentile§	Model	OR	95 % CI	*P*
High adherence *v*. non-adherence	Model 1, *n* 881	0·68	0·42, 1·10	0·12
	Model 2, *n* 881	0·72	0·43, 1·20	0·21
	Model 3, *n* 737	0·80	0·45, 1·42	0·44
High adherence *v*. low adherence	Model 1, *n* 534	0·80	0·31, 2·05	0·64
	Model 2, *n* 534	1·23	0·44, 3·45	0·70
	Model 3, *n* 454	1·91	0·60, 6·15	0·28

* Differences between the adherence groups were calculated using multiple linear regression for continuous outcomes and multiple logistic regression for percentile outcomes.

† High adherence was defined as consuming 10 µg vitamin D supplementation 6–7 times per week during at least 80 % of the observation time, and non-adherent as otherwise. Low adherence was defined as consuming supplementation for 6 months or less. The reference group is the high-adherence group.

‡ Model 1 is adjusted for height (cm) and sex (male/female). Model 2 is adjusted for height (cm), sex (male/female), gestational age (weeks), parity (unipara/multipara), and child BMI (kg/m^2^). Model 3 is adjusted for height (cm), sex (male/female), gestational age (weeks), parity (unipara/multipara), child BMI (kg/m^2^), physical activity (less active than peers/as active as peers/more active than peers), skin tone (Fitzpatrick scale 1–2/3–6), and daily dairy product consumption (<3 dl/d *v*. ≥3 dl/d).

§ >10th percentile (>0.554 g/cm^2^) is the reference group.

**Table 3.** Association between adherence to vitamin D supplementation at the age of 18 months and bone mineral density (BMD) *z*-score, bone mineral content (BMC) (g), and bone area (BA) (cm^2^) at the age of 7 years* (*β*-Coefficients and 95 % confidence intervals)

	Model†	*β*	95 % CI	*P*
TBLH BMD *z*-score				
High adherence‡ *v*. non-adherence	Model 1, *n* 881	0·018	–0·069, 0·106	0·68
	Model 2, *n* 881	–0·003	–0·084, 0·078	0·95
	Model 3, *n* 737	–0·014	–0·100, 0·072	0·76
High adherence *v*. low adherence§	Model 1, *n* 534	0·031	–0·155, 0·216	0·75
	Model 2, *n* 534	–0·060	–0·235, 0·115	0·50
	Model 3, *n* 454	0·007	–0·192, 0·205	0·95
TBLH BMC				
High adherence *v*. non-adherence	Model 1, *n* 881	3·857	–3·370, 11·084	0·30
	Model 2, *n* 881	2·032	–3·686, 7·750	0·49
	Model 3, *n* 737	1·001	–5·171, 7·173	0·75
High adherence *v*. low adherence	Model 1, *n* 534	9·605	–5·592, 24·802	0·22
	Model 2, *n* 534	0·902	–11·543, 13·346	0·89
	Model 3, *n* 454	4·133	–10·176, 18·441	0·57
TBLH BA				
High adherence *v*. non-adherence	Model 1, *n* 881	4·232	–1·888, 10·353	0·18
	Model 2, *n* 881	3·453	–1·831, 8·738	0·20
	Model 3, *n* 737	3·258	–2·577, 9·092	0·27
High adherence *v*. low adherence	Model 1, *n* 534	9·365	–3·627, 22·358	0·18
	Model 2, *n* 534	3·729	–7·515, 14·973	0·52
	Model 3, *n* 454	1·620	–11·654, 14·894	0·81

TBLH, total-body-less-head.

* Differences between the adherence groups were calculated using multiple linear regression.

† Model 1 is adjusted for height (cm) and sex (male/female). Model 2 is adjusted for height (cm), sex (male/female), gestational age (weeks), parity (unipara/multipara) and child BMI (kg/m^2^). Model 3 is adjusted for height (cm), sex (male/female), gestational age (weeks), parity (unipara/multipara), child BMI (kg/m^2^), physical activity (less active than peers/as active as peers/more active than peers), skin tone (Fitzpatrick scale 1–2/3–6) and daily dairy product consumption (<3 dl/d *v*. ≥3 dl/d).

‡ High adherence was defined as consuming 10 µg of vitamin D supplementation 6–7 times per week during at least 80 % of the observation time, and non-adherent as otherwise.

§ Low adherence was defined as consuming supplementation for 6 months or less.

### Associations between serum 25-hydroxyvitamin D concentrations and total-body-less-head bone mineral density

No consistent associations were found between TBLH BMD and s-25(OH)D in early or late pregnancy, in umbilical cord, or at the age of 5 years. These results were consistent using s-25(OH)D on a continuous scale, and when applying either clinical cut-off values or quartiles, as well as when comparing the lowest TBLH BMD 10th percentiles to the remaining values (online Supplementary Tables S1 and S2). As an exception, the odds for having a TBLH BMD ≤ 10th percentile was associated with early pregnancy s-25(OH)D < 25 nmol/l (model 3, adjusted OR 4·82 (1·08, 21·57), *P* = 0·04, *n* 16) compared with those having a TBLH BMD > 10th percentile (online Supplementary Table S2). Applying the non-interacting covariate season of blood sampling in the models weakened this association to some degree (model 3, adjusted OR 4·35 (0·97, 19·58), *P* = 0·06, *n* 16). Applying season of blood sampling and/or season of questionnaire answering (data on vitamin D supplementation) did not change any other results at any time point (results not shown).

### Recent vitamin D supplementation and total-body-less-head bone mineral density

Looking at vitamin D supplementation intake at the ages of 5 and 7 years by comparing those using supplementation twice per week or more with those using less, the basic models suggested inverse associations between the use of vitamin D supplementation at the age of 7 years and TBLH BMD, BMC and BMD *z*-score at the age of 7 years. However, these inverse associations disappeared when adjusting for additional variables in model 2 and model 3. At the age of 5 years, no associations were found (online Supplementary Table S3).

### Other covariates

In a backwards multiple linear regression analysis, TBLH BMD was positively associated with the current child height, child BMI, physical activity and dairy product consumption. Season at examination, parity, child sex, skin tone and gestational age showed no associations (results not shown).

### Differences between participants included in regression model 1 and model 3

For the main analysis, model 1 included 881 participants. Inclusion of more covariates in model 3 resulted in the exclusion of 144 participants due to missing values. When comparing the 144 excluded participants with the included 737 participants in model 3, no differences were seen for vitamin D supplementation at any age, s-25(OH)D at any age, DXA results or in any other variables included in the models. However, the non-included mothers were younger and more often smoked during pregnancy, had lower educational level, were more likely to have answered the 18-month questionnaire between November and April, and had children who were slightly older at the physical examination at the age of 7 years (online Supplementary Table S4).

### Participants and non-participants

In comparison with our participants, non-participant OCC children had a shorter body length at birth, were exclusively breastfed for shorter time periods and were less likely to have older siblings. The mothers were younger and had a lower educational level but had higher levels of s-25(OH)D in early pregnancy (online Supplementary Table S5). No difference was seen for adherence to vitamin D supplementation recommendations in infancy.

## Discussion

In this population-based cohort of children and their mothers, no consistent associations were found between vitamin D supplementation use at the ages of 18 months, 5 years and 7 years and TBLH BMD, BMC or BA measured by DXA scan at the age of 7 years. Similarly, no consistent associations were found between s-25(OH)D in early pregnancy, late pregnancy, umbilical cord or at the age of 5 years and TBLH BMD. Our population was representative for the background population cohort with regard to adherence to vitamin D supplementation in infants and the other covariates in the models but differed with regard to age, education level, s-25(OH)D in early pregnancy, and parity of the mothers as well as body length at birth, weeks of exclusive breast-feeding, and holidays of the child and may well differ compared with other populations. For example, the vitamin D status in our study population was generally high, from pregnancy to the age of 5 years. Of note, no participants in the entire OCC had rickets. The value of vitamin D supplementation in pregnancy and infancy to prevent rickets, as confirmed in consensus guidelines^(6)^, was not questioned by our findings.

Our results regarding supplementation in infancy and BMD are compatible with three RCTs^(23,24,41,42)^ investigating the effects of receiving 30 or 40 µg of vitamin D supplementation daily in infancy compared with the recommended 10 µg. None of these studies found significant differences in BMD at follow-up between the ages of 3 months and 3 years. Our study suggests that even lower doses of vitamin D supplementation may be sufficient in a well-off, well-nourished population.

Furthermore, our study suggests that 10 µg of daily vitamin D supplementation up to 18 months (compared with less supplementation) has no conditioning effect on BMD later in childhood. A plausible explanation of this finding is that even the low-adherence group in our study may have sufficient s-25(OH)D concentrations due to dietary intake and sun exposure to support an optimal BMD. This is possible because the typical Danish diet is rich in Ca^(43,44)^, whereby even a lower fractional Ca absorption caused by a suboptimal s-25(OH)D may still result in an overall sufficient Ca uptake^(45)^. Indeed, dairy product consumption was associated with 7-year BMD in the backwards regression. Alternatively, vitamin D supplementation in infancy may temporarily improve bone mineralisation during the first years of life, but without sustained effects.

We did not find any robust associations between s-25(OH)D in pregnancy or cord blood and bone mineralisation. The association between s-25(OH)D < 25 nmol/l in early pregnancy (*n* 16) and TBLH BMD in the lowest 10th percentile weakened in sensitivity analyses after including season in the regression models.

Our general null findings are in line with findings from a large RCT where 25 µg of daily supplementation in pregnancy *v*. placebo did not affect newborn total body BMD, BMC and BA^(16)^, and with two large cohort studies observing no associations between s-25(OH)D in pregnancy and offspring bone mineralisation^(12,46)^. Smaller observational studies have observed direct associations^(11,47)^, including the recent Australian cohort study, where s-25(OH)D < 28 nmol/l (*n* 12) in early pregnancy was associated with TBLH BMD in boys, but not in girls^(47)^. A large observational study (*n* 3960), however, failed to detect any associations between s-25(OH)D in any trimester of pregnancy and offspring TBLH BMC at 9·9 years^(12)^. Taken together, the evidence does not support a conditioning role of early or late pregnancy vitamin D status on offspring BMD. This should, however, not be extrapolated to populations with a higher prevalence of vitamin D deficiency.

Brustad *et al.*^(17)^ randomised pregnant women to different doses of fish oil and to 70 *v*. 10 µg of vitamin D daily in the third trimester. In a secondary analysis, a direct effect on TBLH BMC and head BMD by DXA at 3 and 6 years was found, but no difference in the more important outcome TBLH BMD^(17)^. We did not observe any associations for the highest s-25(OH)D concentrations and DXA TBLH BMD, BMC or BA, which, however, does not rule out effects of very high-dose vitamin D supplementation. Potential adverse effects of high-dose vitamin D supplementation should, however, be taken into account when evaluating recommendations on vitamin D supplementation.

We found no consistent associations between more recent vitamin D supplementation (i.e. at 5 and 7 years) or 25(OH)D concentrations and TBLH BMD, BMC or BA. This is in line with the results of several other studies performed in the Nordic countries finding no effect of recent vitamin D supplementation on BMD or BMC in RCTs^(21,22)^, and no associations to recent or current s-25(OH)D in observational studies^(48,49)^. In other countries, results are more diverse^(19,20,50,51)^. Both genetic factors as well as dietary intake of Ca, vitamin D, salts, and protein, sun exposure, physical activity, and other lifestyle factors may explain these differences. Our results suggest that recent or current s-25(OH)D concentrations above 50 nmol/l are sufficient to support optimal bone health in children, as seen for adults according to the Institute of Medicine^(26)^. For the 20 % of the children with s-25(OH)D < 50 nmol/l at 5 years, other dietary and lifestyle factors may have compensated to ensure a normal bone mineralisation, as bone mineralisation in children is not dependent of vitamin D status alone, but the combined result of dietary intake of Ca and vitamin D status^(45)^.

The Danish Health Authorities recommended at the time of the study daily supplementation of 10 µg of vitamin D in pregnancy and from the age of 2 weeks to 2 years to all children. The adherence to the vitamin D guideline recommendations was high in just above 50 % of participants, but similar or lower adherence has also been detected in recent Irish^(30)^ and American^(29)^ studies. We observed several well-known risk factors for low adherence to vitamin D supplementation recommendations for children up to the age of 18 months, but also higher parity as a novel risk factor. This is in line with our previous demonstration of higher parity as a risk factor of low vitamin D status in pregnancy^(32)^. Some studies have found higher parity to be associated with lower vitamin D status or vitamin D intake in pregnancy^(52–55)^, while others have found the contrary^(56–58)^. In our previous OCC studies, higher parity correlated strongly with both lower adherence to vitamin D supplementation recommendations and with lower s-25(OH)D in pregnancy and cord blood^(32,59)^. These results indicate that the differences in vitamin D status between unipara and multipara may be caused by behavioural or social factors rather than having a biological explanation. We speculate that similar factors, for example, variations in perfectionism and domestic workload, may explain the lower adherence to vitamin D supplementation guidelines in infants with increasing parity. Of note, parity was an independent risk factor, ruling out parity as a proxy for maternal age and educational status.

Despite a relatively low adherence to the vitamin D supplementation recommendations evaluated at 18 months, no children had rickets, and no differences were found in TBLD BMD or other bone parameters, or anthropometric measures in our cohort. Furthermore, no differences were seen between children who never used vitamin D supplementation or used supplementation for ≤6 months compared with those still using vitamin D supplement as recommended by 18 months of age. This suggests a robustness of bone health regardless of inferior adherence to vitamin D supplementation in well-nourished children living in developed countries, even at higher latitudes. On the other hand, no inverse associations were found to argue against the national recommendations. To our best knowledge, no existing high-quality data support a praxis of vitamin D supplementation in children beyond 12 months of age, except in risk groups.

### Strengths and limitations

Strengths of the present study include its relatively large size as well as the population-based prospective design; information on vitamin D supplementation repeatedly collected through questionnaires thereby limiting the risk of recall bias; and s-25(OH)D measurements performed by the ‘gold standard’ method at various times from early pregnancy to the age of 5 years. Additionally, the inclusion of information on lifestyle, such as dairy product consumption, physical activity and physical features such as skin tone, height, sex and BMI strengthens the study. Furthermore, our choice of outcome variables follows the consensus guidelines for interpretation of DXA scans in children^(60)^.

A limitation of the present study is its observational nature including inability to draw conclusions regarding causality of findings and risk of residual confounding. Other limitations include risk of selection bias from the background population and the risk of information bias in questionnaires. Furthermore, data were not available on bone mineralisation in infancy, s-25(OH)D at the age of 7 years, sun exposure and nutritional vitamin D intake. However, vitamin D fortification of food was not common in Denmark at the time studied. S-25(OH)D was not measured in the same child at various seasons; therefore, we cannot ensure that these values are representative for the individual status year round. Lastly, our findings may have limited external validity, especially for other populations with differences in, for example, ethnicity, diet, sun exposure, vitamin D supplementation and prevalence of vitamin D deficiency.

### Conclusion

In the population-based OCC, 53·9 % of the participants were highly adherent to the national guidelines on vitamin D supplementation in infancy. No associations were found between vitamin D supplementation use at the ages of 18 months, 5 years and 7 years and TBLH BMD, BMC or BA measured by DXA scan at the age of 7 years. Similarly, s-25(OH)D in early pregnancy, late pregnancy, umbilical cord or at the age of 5 years was not robustly associated with TBLH BMD. Adverse bone effects of low adherence to vitamin D supplementation guidelines in infancy, or low vitamin D status in pregnancy and childhood, do not seem to be of major concern in the population studied.

## Supplementary material

For supplementary material accompanying this paper visit https://doi.org/10.1017/S0007114521000301. click here to view supplementary material
