# Supplementary material for: Bone mineral density at age 7 years does not associate with adherence to vitamin D supplementation guidelines in infancy or vitamin D status in pregnancy and childhood: an Odense Child Cohort study
Source: Br J Nutr. 2021 Jan 26;126(10):1466–77. doi: 10.1017/S0007114521000301 (PMC8524427; doi:10.1017/S0007114521000301)
Supplement: Supplementary file 1 [file S0007114521000301sup001.docx]

**Supplementary material**

| **Supplementary Table 1:** Association between s-25(OH)D in early pregnancy, late pregnancy, umbilical cord, and at age 5 years, and continuous TBLH BMD (g/cm^2^). | | | | | |
| --- | --- | --- | --- | --- | --- |
|  | S-25(OH)D (nmol/l) | Beta Coefficient | 95 % C.I. | | *p*-value |
| *Early pregnancy** | | | | | |
| Continuous s-25(OH)D |  |  |  |  |  |
| Model 1, *n =* 600 |  | −0.000 | (−0.000; 0.000) | | 0.12 |
| Model 2, *n =* 600 |  | −0.000 | (−0.000; 0.000) | | 0.76 |
| Model 3, *n =* 472 |  | −0.000 | (−0.000; 0.000) | | 0.59 |
| Quartiles of s-25(OH)D† |  |  |  |  |  |
| Model 1, *n =* 600 | Q1 [8.47, 49.10] | 0.009 | (0.000; 0.019) | | **0.04** |
|  | Q2 [49.11, 64.65] | −0.000 | (−0.009; 0.009) | | 0.95 |
|  | Q3 [64.75, 77.41] | 0.000 | (−0.009; 0.009) | | 0.97 |
| Model 2, *n =* 600 | Q1 | 0.004 | (−0.005; 0.012) | | 0.39 |
|  | Q2 | −0.002 | (−0.010; 0.006) | | 0.67 |
|  | Q3 | −0.001 | (−0.009; 0.0077) | | 0.90 |
| Model 3, *n =* 472 | Q1 | 0.003 | (−0.006; 0.012) | | 0.49 |
|  | Q2 | 0.001 | (−0.008; 0.010) | | 0.83 |
|  | Q3 | −0.001 | (−0.010; 0.008) | | 0.84 |
| Clinical cuts of s-25(OH)D‡ |  |  |  |  |  |
| Model 1, *n =* 600 | < 25 | −0.005 | (−0.026; 0.015) | | 0.62 |
|  | 25–50 | 0.010 | (0.001; 0.018) | | **0.04** |
|  | 50–75 | 0.001 | (−0.007; 0.009) | | 0.80 |
| Model 2, *n =* 600 | < 25 | −0.012 | (−0.031; 0.006) | | 0.19 |
|  | 25–50 | 0.004 | (−0.004; 0.012) | | 0.37 |
|  | 50–75 | −0.001 | (−0.008; 0.006) | | 0.74 |
| Model 3, *n =* 472 | < 25 | −0.014 | (−0.034; 0.005) | | 0.15 |
|  | 25–50 | 0.004 | (−0.004; 0.013) | | 0.32 |
|  | 50–75 | −0.001 | (−0.008; 0.007) | | 0.90 |
| *Late pregnancy*§ | | | | | |
| Continuous s-25(OH)D |  |  |  |  |  |
| Model 1, *n =* 679 |  | −0.000 | (−0.000; 0.000) | | 0.09 |
| Model 2, *n =* 679 |  | −0.000 | (−0.000; 0.000) | | 0.31 |
| Model 3, *n =* 546 |  | −0.000 | (−0.000; 0.000) | | 0.14 |
| Quartiles of s-25(OH)D† |  |  |  |  |  |
| Model 1, *n =* 679 | Q1 [10.02, 60.42] | 0.007 | (−0.002; 0.015) | | 0.14 |
|  | Q2 [60.53, 78.82] | 0.005 | (−0.004; 0.013) | | 0.27 |
|  | Q3 [79.20, 96.35] | 0.005 | (−0.004; 0.013) | | 0.30 |
| Model 2, *n =* 679 | Q1 | 0.003 | (−0.005; 0.011) | | 0.47 |
|  | Q2 | 0.003 | (−0.005; 0.011) | | 0.47 |
|  | Q3 | 0.001 | (−0.007; 0.009) | | 0.78 |
| Model 3, *n =* 546 | Q1 | 0.006 | (−0.003; 0.014) | | 0.19 |
|  | Q2 | 0.001 | (−0.008; 0.009) | | 0.88 |
|  | Q3 | 0.004 | (−0.005; 0.013) | | 0.36 |
| Clinical cuts s-25(OH)D‡ |  |  |  |  |  |
| Model 1, *n =* 679 | < 25 | 0.002 | (−0.019; 0.022) | | 0.87 |
|  | 25–50 | 0.003 | (−0.006; 0.012) | | 0.54 |
|  | 50–75 | 0.001 | (−0.006; 0.008) | | 0.76 |
| Model 2, *n =* 679 | < 25 | 0.001 | (−0.017; 0.019) | | 0.91 |
|  | 25–50 | 0.002 | (−0.006; 0.010) | | 0.65 |
|  | 50–75 | 0.001 | (−0.006; 0.007) | | 0.85 |
| Model 3, *n =* 546 | < 25 | 0.002 | (−0.018; 0.022) | | 0.83 |
|  | 25–50 | 0.003 | (−0.006; 0.012) | | 0.50 |
|  | 50–75 | −0.000 | (−0.007; 0.007) | | 0.90 |
| *Umbilical cord* |  |  |  |  |  |
| Continuous of s-25(OH)D |  |  |  |  |  |
| Model 1, *n =* 997 |  | 7.57∙10^−6^ | (−0.000; 0.000) | | 0.90 |
| Model 2, *n =* 941 |  | 0.0000 | (−0.000; 0.000) | | 0.55 |
| Model 3, *n =* 797 |  | −0.0000 | (−0.000; 0.000) | | 0.85 |
| Quartiles of s-25(OH)D† |  |  |  |  |  |
| Model 1, *n =* 997 | Q1 [1.53, 31.35] | 0.0005 | (−0.007; 0.008) | | 0.89 |
|  | Q2 [31.52, 45.97] | −0.0026 | (−0.010; 0.004) | | 0.47 |
|  | Q3 [46.04, 61.08] | 0.0025 | (−0.005; 0.009) | | 0.53 |
| Model 2, *n =* 997 | Q1 | −0.0014 | (−0.008; 0.005) | | 0.66 |
|  | Q2 | −0.0014 | (−0.008; 0.005) | | 0.67 |
|  | Q3 | 0.0005 | (−0.006; 0.007) | | 0.89 |
| Model 3, *n =* 797 | Q1 | 0.0010 | (−0.006; 0.008) | | 0.79 |
|  | Q2 | −0.0004 | (−0.007; 0.007) | | 0.91 |
|  | Q3 | 0.0010 | (−0.006; 0.008) | | 0.78 |
| Clinical cuts of s-25(OH)D\|\| |  |  |  |  |  |
| Model 1, *n =* 997 | < 25 | −0.0012 | (−0.009; 0.006) | | 0.76 |
|  | 25–50 | −0.0023 | (−0.008; 0.004) | | 0.45 |
|  | > 75 | −0.0003 | (−0.009; 0.008) | | 0.94 |
| Model 2, *n =* 997 | < 25 | −0.0015 | (−0.008; 0.005) | | 0.67 |
|  | 25–50 | −0.0012 | (−0.006; 0.004) | | 0.65 |
|  | > 75 | 0.0027 | (−0.005; 0.010) | | 0.50 |
| Model 3, *n =* 797 | < 25 | 0.0015 | (−0.006; 0.009) | | 0.69 |
|  | 25–50 | −0.0012 | (−0.007; 0.005) | | 0.69 |
|  | > 75 | 0.0026 | (−0.006; 0.011) | | 0.55 |
| *Age 5 years* |  |  |  |  |  |
| Continuous of s-25(OH)D |  |  |  |  |  |
| Model 1, *n =* 658 |  | −0.000 | (−0.000; 0.000) | | 0.42 |
| Model 2, *n =* 658 |  | −0.000 | (−0.000; 0.000) | | 0.30 |
| Model 3, *n =* 530 |  | −0.000 | (−0.000; 0.000) | | 0.22 |
| Quartiles of s-25(OH)D† |  |  |  |  |  |
| Model 1, *n =* 658 | Q1 [6.80, 53.90] | 0.003 | (−0.006; 0.012) | | 0.46 |
|  | Q2 [53.90, 71.77] | −0.000 | (−0.009; 0.009) | | 0.99 |
|  | Q3 [71.89, 86.72] | −0.003 | (−0.012; 0.006) | | 0.51 |
| Model 2, *n =* 658 | Q1 | 0.005 | (−0.003; 0.013) | | 0.22 |
|  | Q2 | 0.001 | (−0.007; 0.009) | | 0.74 |
|  | Q3 | −0.001 | (−0.009; 0.007) | | 0.84 |
| Model 3, *n =* 530 | Q1 | 0.006 | (−0.003; 0.015) | | 0.16 |
|  | Q2 | 0.000 | (−0.008; 0.009) | | 0.92 |
|  | Q3 | −0.003 | (−0.011; 0.006) | | 0.51 |
| Clinical cuts of s-25(OH)D‡ |  |  |  |  |  |
| Model 1, *n =* 658 | < 25 | −0.003 | (−0.025; 0.018) | | 0.75 |
|  | 25–50 | 0.005 | (−0.004; 0.014) | | 0.29 |
|  | 50–75 | −0.000 | (−0.007; 0.007) | | 0.97 |
| Model 2, *n =* 658 | < 25 | 0.001 | (−0.018; 0.020) | | 0.92 |
|  | 25–50 | 0.005 | (−0.003; 0.013) | | 0.20 |
|  | 50–75 | 0.001 | (−0.005; 0.007) | | 0.79 |
| Model 3, *n =* 530 | < 25 | −0.002 | (−0.025; 0.020) | | 0.84 |
|  | 25–50 | 0.008 | (−0.001; 0.016) | | 0.08 |
|  | 50–75 | 0.002 | (−0.005; 0.008) | | 0.66 |
| Abbreviations: s-25(OH)D, serum 25-hydroxyvitamin D; TBLH BMD, total-body-less-head bone mineral density; C.I., confidence interval.  *Early pregnancy s-25(OH)D is measured in blood samples collected before week 20 of pregnancy.  †The reference group at all time points is those in the highest s-25(OH) quartile.  ‡The reference group is those with s-25(OH)D > 75 nmol/l.  §Late pregnancy s-25(OH)D is measured in blood samples collected after week 20 of pregnancy.  \|\|The reference group is those with s-25(OH)D 50-75 nmol/l.  Model 1 is adjusted for height (cm) and sex (male/female). Model 2 is adjusted for height (cm), sex (male/female), gestational age (weeks), parity (unipara/multipara) and child BMI (kg/m^2^). Model 3 is adjusted for height (cm), sex (male/female), gestational age (weeks), parity (unipara/multipara), child BMI (kg/m^2^), physical activity (less active than peers/as active as peers/more active than peers), skin tone (Fitzpatrick scale 1-2/3-6), and daily dairy consumption (< 3 dl/day vs. ≥ 3 dl/day). Outcomes were calculated using multiple linear regression. | | | | | |

| **Supplementary Table 2:** Association between s-25(OH)D in early pregnancy, late pregnancy, umbilical cord, and age 5 years, and TBLH BMD (g/cm^2^) ≤ 10^th^ vs. > 10^th^ percentile (reference group*). | | | | | | |
| --- | --- | --- | --- | --- | --- | --- |
|  | S-25(OH)D (nmol/l) | Odds ratio | 95 % C.I. | *p*-value | | |
| *Early pregnancy*† |  |  |  |  | | |
| Continuous |  |  |  |  | | |
| Model 1, *n =* 600 |  | 0.70 | (0.98; 1.01) | 0.55 | | |
| Model 2, *n =* 600 |  | 0.99 | (0.98; 1.01) | 0.30 | | |
| Model 3, *n =* 472 |  | 0.99 | (0.97; 1.01) | 0.21 | | |
| Quartiles of s-25(OH)D‡ |  |  |  |  | | |
| Model 1, *n =* 600 | Q1 [8.47, 49.10] | 0.79 | (0.34; 1.84) | 0.59 | | |
|  | Q2 [49.11, 64.65] | 1.17 | (0.55; 2.56) | 0.68 | | |
|  | Q3 [64.75, 77.41] | 0.52 | (0.22; 1.23) | 0.14 | | |
| Model 2, *n =* 600 | Q1 | 1.04 | (0.42; 2.56) | 0.94 | | |
|  | Q2 | 1.21 | (0.55; 2.67) | 0.64 | | |
|  | Q3 | 0.52 | (0.22; 1.26) | 0.15 | | |
| Model 3, *n =* 472 | Q1 | 1.28 | (0.47; 3.47) | 0.63 | | |
|  | Q2 | 1.34 | (0.56; 3.24) | 0.51 | | |
|  | Q3 | 0.51 | (0.19; 1.39) | 0.19 | | |
| Clinical cuts of s-25(OH)D§ |  |  |  |  | | |
| Model 1, *n =* 600 | < 25 | 3.03 | (0.87; 10.53) | 0.08 | | |
|  | 25–50 | 0.67 | (0.27; 1.68) | 0.40 | | |
|  | > 75 | 1.30 | (0.67; 2.55) | 0.44 | | |
| Model 2, *n =* 600 | < 25 | 4.89 | (1.24; 19.25) | **0.023** | | |
|  | 25–50 | 0.69 | (0.26; 1.82) | 0.45 | | |
|  | > 75 | 1.14 | (0.57; 2.28) | 0.70 | | |
| Model 3, *n =* 472 | < 25 | 4.82 | (1.08; 21.57) | **0.040** | | |
|  | 25–50 | 0.90 | (0.32; 2.56) | 0.84 | | |
|  | > 75 | 1.03 | (0.47; 2.23) | 0.95 | | |
| *Late pregnancy*\|\| | | | | | | |
| Continuous s-25(OH)D | | | | | | |
| Model 1, *n =* 679 |  | 1.01 | (1.00; 1.02) | 0.31 | | |
| Model 2, *n =* 679 |  | 1.00 | (0.99; 1.01) | 0.81 | | |
| Model 3, *n =* 546 |  | 1.00 | (0.99; 1.01) | 0.91 | | |
| Quartiles§ of s-25(OH)D‡ | | | | | | |
| Model 1, *n =* 679 | Q1 [10.02, 60.42] | 0.60 | (0.26; 1.41) | 0.24 | | |
|  | Q2 [60.53, 78.82] | 1.26 | (0.60; 1.41) | 0.54 | | |
|  | Q3 [79.20, 96.35] | 1.23 | (0.59; 2.57) | 0.59 | | |
| Model 2, *n =* 679 | Q1 | 0.78 | (0.32; 1.90) | 0.58 | | |
|  | Q2 | 1.52 | (0.70; 3.32) | 0.29 | | |
|  | Q3 | 1.53 | (0.71; 3.31) | 0.28 | | |
| Model 3, *n =* 546 | Q1 | 0.78 | (0.29; 2.31) | 0.63 | | |
|  | Q2 | 1.61 | (0.69; 3.80) | 0.27 | | |
|  | Q3 | 1.21 | (0.51; 2.86) | 0.66 | | |
| Clinical cuts of s-25(OH)D§ | | | | | | |
| Model 1, *n =* 679 | < 25 | 0.64 | (0.08; 5.28) | 0.67 | | |
|  | 25–50 | 0.46 | (1.16; 1.34) | 0.16 | | |
|  | > 75 | 0.99 | (0.54; 1.82) | 0.98 | | |
| Model 2, *n =* 679 | < 25 | 0.65 | (0.08; 5.72) | 0.70 | | |
|  | 25–50 | 0.58 | (0.19; 1.72) | 0.32 | | |
|  | > 75 | 0.95 | (0.51; 1.80) | 0.89 | | |
| Model 3, *n =* 546 | < 25 | 0.85 | (0.09; 8.30) | 0.89 | | |
|  | 25–50 | 0.60 | (0.18; 2.02) | 0.41 | | |
|  | > 75 | 0.82 | (0.40; 1.67) | 0.58 | | |
| *Umbilical cord* | | | | | | |
| Continuous s-25(OH)D | | | | | | |
| Model 1, *n =* 997 |  | 1.01 | (1.00; 1.02) | | 0.30 | |
| Model 2, *n =* 941 |  | 1.01 | (1.00; 1.02) | | 0.29 | |
| Model 3, *n =* 797 |  | 1.00 | (1.00; 1.01) | | 0.82 | |
| Quartiles of s-25(OH)D | | | | | | |
| Model 1, *n =* 997 | Q1 [1.53, 31.35] | 0.79 | (0.41; 1.50) | | 0.47 | |
|  | Q2 [31.52, 45.97] | 0.97 | (0.54; 1.77) | | 0.93 | |
|  | Q3 [46.04, 61.08] | 0.78 | (0.42; 1.45) | | 0.44 | |
| Model 2, *n =* 997 | Q1 | 0.84 | (0.42; 1.67) | | 0.62 | |
|  | Q2 | 0.95 | (0.51; 1.75) | | 0.86 | |
|  | Q3 | 0.88 | (0.46; 1.67) | | 0.69 | |
| Model 3, *n =* 797 | Q1 | 1.26 | (0.58; 2.71) | | 0.56 | |
|  | Q2 | 1.05 | (0.52; 2.10) | | 0.90 | |
|  | Q3 | 1.07 | (0.51; 2.26) | | 0.86 | |
| Clinical cuts of s-25(OH)D | | | | |  |  |
| Model 1, *n =* 997 | < 25 | 0.67 | (0.30; 1.49) | | 0.33 | |
|  | 25–50 | 0.11 | (0.66; 1.87) | | 0.69 | |
|  | > 75 | 0.24 | (0.61; 2.55) | | 0.55 | |
| Model 2, *n =* 997 | < 25 | 0.63 | (0.27; 1.45) | | 0.28 | |
|  | 25–50 | 1.07 | (0.62; 1.84) | | 0.81 | |
|  | > 75 | 1.12 | (0.53; 2.38) | | 0.76 | |
| Model 3, *n =* 797 | < 25 | 0.96 | (0.39; 2.34) | | 0.92 | |
|  | 25–50 | 1.27 | (0.68; 2.37) | | 0.45 | |
|  | > 75 | 1.23 | (0.53; 2.85) | | 0.63 | |
| *Age 5 years* | | | | | | |
| Continuous of s-25(OH)D | | | | | | |
| Model 1, *n =* 658 |  | 1.01 | (1.00; 1.02) | | 0.09 | |
| Model 2, *n =* 658 |  | 1.01 | (1.00; 1.02) | | 0.14 | |
| Model 3, *n =* 530 |  | 1.01 | (1.01; 1.02) | | 0.57 | |
| Quartiles of s-25(OH) | | | | | | |
| Model 1, *n =* 658 | Q1 [6.80, 53.90] | 0.46 | (0.21; 1.05) | | 0.07 | |
|  | Q2 [53.90, 71.77] | 0.69 | (0.33; 1.44) | | 0.32 | |
|  | Q3 [71.89, 86.72] | 0.84 | (0.41; 1.71) | | 0.62 | |
| Model 2, *n =* 658 | Q1 | 0.44 | (0.18; 1.03) | | 0.06 |  |
|  | Q2 | 0.79 | (0.36; 1.73) | | 0.55 |  |
|  | Q3 | 0.80 | (0.37; 1.73) | | 0.58 |  |
| Model 3, *n =* 530 | Q1 | 0.56 | (0.22; 1.42) | | 0.22 |  |
|  | Q2 | 0.69 | (0.27; 1.72) | | 0.42 |  |
|  | Q3 | 0.91 | (0.38; 2.17) | | 0.84 |  |
| Clinical cuts of s-25(OH)D | | | | |  |  |
| Model 1, *n =* 658 | < 25 | 1.44 | (0.25; 8.33) | | 0.68 |  |
|  | 25–50 | 0.59 | (0.22; 1.56) | | 0.28 |  |
|  | > 75 | 1.41 | (0.76; 2.60) | | 0.28 |  |
| Model 2, *n =* 658 | < 25 | 0.91 | (0.15; 5.68) | | 0.92 |  |
|  | 25–50 | 0.55 | (0.20; 1.51) | | 0.25 |  |
|  | > 75 | 1.30 | (0.68; 2.48) | | 0.43 |  |
| Model 3, *n =* 530 | < 25 | 2.67 | (0.30; 23.32) | | 0.38 |  |
|  | 25–50 | 0.94 | (0.32; 2.80) | | 0.91 |  |
|  | > 75 | 1.82 | (0.85; 3.91) | | 0.12 |  |
| Abbreviations: s-25(OH)D, serum 25-hydroxyvitamin D; TBLH BMD; total-body-less-head bone mineral density; C.I., confidence interval.  *10^th^ percentile BMD: Early pregnancy = 0.554 g/cm^2^; late pregnancy = 0.556 g/cm^2^; umbilical cord = 0.556 g/cm^2^; 5 years = 0.554 g/cm^2^.  †Early-pregnancy s-25(OH)D is measured in blood samples collected before week 20 of pregnancy.  ‡Reference group is those within the highest s-25(OH)D quartile at all time points.  §Reference group is those with s-25(OH)D 50-75 nmol/l.  \|\|Late-pregnancy s-25(OH)D is measured in blood samples collected after week 20 of pregnancy.  Model 1 is adjusted for height (cm) and sex (male/female). Model 2 is adjusted for height (cm), sex (male/female), gestational age (weeks), parity (unipara/multipara) and child BMI (kg/m^2^). Model 3 is adjusted for height (cm), sex (male/female), gestational age (weeks), parity (unipara/multipara), child BMI (kg/m^2^), physical activity (less active than peers/as active as peers/more active than peers), skin tone (Fitzpatrick scale 1-2/3-6), and daily dairy consumption (< 3 dl/day vs. ≥ 3 dl/day). Outcomes were calculated using logistic regression. | | | | | |  |

| **Supplementary Table 3:** Association between vitamin D supplementation at age 5 and 7 years, and BMD (g/cm^2^), BMD z-score, BMD (g), and BA (cm^2^). | | | | | |
| --- | --- | --- | --- | --- | --- |
|  | Model* | Beta Coefficient | 95 % C.I. | | *p-*value |
| *Supplement age 5*† |  |  |  |  |  |
| TBLH BMD | Model 1, *n =* 960 | 0.004 | (−0.001; 0.009) | | 0.15 |
|  | Model 2, *n =* 960 | 0.002 | (−0.003; 0.006) | | 0.41 |
|  | Model 3, *n =* 816 | −0.002 | (−0.007; 0.003) | | 0.42 |
| TBLH BMD | Model 1, *n =* 960 | 0.057 | (−0.027; 0.140) | | 0.18 |
| *z*-score | Model 2, *n =* 960 | 0.027 | (−0.047; 0.101) | | 0.47 |
|  | Model 3, *n =* 816 | 0.029 | (−0.049; 0.107) | | 0.47 |
| TBLH BMC | Model 1, *n =* 960 | 6.417 | (−0.439; 13.274) | | 0.07 |
|  | Model 2, *n =* 960 | 3.295 | (−1.920; 8.511) | | 0.22 |
|  | Model 3, *n =* 816 | 3.484 | (−2.055; 9.023) | | 0.22 |
| TBLH BA | Model 1, *n =* 960 | 4.354 | (−1.350; 10.06) | | 0.13 |
|  | Model 2, *n =* 960 | 2.387 | (−2.348; 7.123) | | 0.32 |
|  | Model 3, *n =* 816 | 2.036 | (−3.185; 7.256) | | 0.44 |
| *Supplement age 7*† |  |  |  |  |  |
| TBLH BMD | Model 1, *n =* 1,021 | 0.006 | (0.001; 0.011) | | **0.02** |
|  | Model 2, *n =* 1,021 | 0.002 | (−0.002; 0.007) | | 0.29 |
|  | Model 3, *n =* 952 | 0.002 | (−0.002; 0.007) | | 0.31 |
| TBLH BMD | Model 1, *n =* 1,021 | 0.096 | (0.0130; 0.180) | | **0.02** |
| *z*-score | Model 2, *n =* 1,021 | 0.034 | (−0.040; 0.108) | | 0.37 |
|  | Model 3, *n =* 952 | 0.031 | (−0.045; 0.107) | | 0.42 |
| TBLH BMC | Model 1, *n =* 1,021 | 9.705 | (2.876; 16.534) | | **0.005** |
|  | Model 2, *n =* 1,021 | 2.915 | (−2.323; 8.153) | | 0.28 |
|  | Model 3, *n =* 952 | 2.441 | (−2.934; 7.816) | | 0.37 |
| TBLH BA | Model 1, *n =* 1,021 | 5.817 | (0.177; 11.457) | | **0.04** |
|  | Model 2, *n =* 1,021 | 1.061 | (−3.687; 5.810) | | 0.66 |
|  | Model 3, *n =* 952 | 0.225 | (−4.730; 5.179) | | 0.93 |
| Abbreviations: BMD, bone mineral density; BMC, bone mineral content; BA, bone area; C.I., confidence interval; TBLH, total-body-less-head  *Model 1 is adjusted for height (cm) and sex (male/female). Model 2 is adjusted for height (cm), sex (male/female), gestational age (weeks), parity (unipara/multipara) and child BMI (kg/m^2^). Model 3 is adjusted for height (cm), sex (male/female), gestational age (weeks), parity (unipara/multipara), child BMI (kg/m^2^), physical activity (less active than peers/as active as peers/more active than peers), skin tone (Fitzpatrick scale 1-2/3-6), and daily dairy consumption (< 3 dl/day vs. ≥ 3 dl/day).  †Consuming vitamin D supplementation is defined as consuming supplementation 2 days/week or more (reference group). Data on vitamin D supplementation in pregnancy and at ages 5 and 7 years could not be specified in more details with regards to doses.  Differences between the groups were calculated using multiple linear regression. | | | | | |

| **Supplementary Table 4:** Descriptive characteristics of participants with incomplete covariate data (participants excluded in regression Model 3) and complete covariate data set (participants in Model 1). | | | | |
| --- | --- | --- | --- | --- |
| **Variable** | **All**  *n* = 881 | **Incomplete data**  *n* = 144 | **Complete data**  *n* = 737 | ***p-*values** |
| Mother’s age at delivery, years, *n* = 881 | 31 [6] | 30 [6] | 31 [6] | **0.04** |
| Mother’s parity *n* = 881  Unipara*, *n* (%) | 464 (52.7 %) | 80 (55.6 %) | 384 (52.1 %) | 0.44 |
| Maternal education, *n* = 876  Lower, *n* (%)  Intermediate, *n* (%)  Higher, *n* (%) | 232 (26.5 %)  437 (49.9 %)  207 (23.6 %) | 53 (37.6 %)  57 (40.4 %)  31 (22.0 %) | 179 (24.4 %)  380 (51.7 %)  176 (24.0 %) | **0.004** |
| Smoking in pregnancy, *n* = 876  Yes, *n* (%) | 38 (4.3 %) | 13 (9.0%) | 25 (3.4 %) | **0.003** |
| Alcohol in pregnancy, *n* = 594  Yes, *n* (%) | 74 (12.5 %) | 9 (9.9 %) | 65 (12.9 %) | 0.42 |
| Vitamin D suppl. in pregnancy†, *n* = 474  Yes *n* (%) | 406 (85.7 %) | 67 (88.2 %) | 339 (85.2 %) | 0.50 |
| s-25(OH)D early preg, nmol/l | 65.8 [28.5] | 65.7 [29.5] | 65.8 [28.4] | 0.80 |
| s-25(OH)D late preg, nmol/l | 78.5 [36.0] | 77.5 [38.1] | 78.6 [38.1] | 0.46 |
| Sex, *n* = 881  Male *n* (%) | 458 (52.0 %) | 66 (45.8 %) | 392 (53.2 %) | 0.11 |
| Gestation age at birth, weeks, *n* = 881 | 40.1 [1.7] | 40.4 [1.7] | 40.1 [1.9] | 0.31 |
| Body weight at birth, g, *n* = 881 | 3555 [675] | 3616 [712] | 3550 [655] | 0.21 |
| Body length at birth, cm, *n* = 874 | 52 [3] | 52 [3] | 52[3] | 0.77 |
| s-25(OH)D cord, nmol/l |  |  |  |  |
| Skintone, Fitzpatrick scale, *n* = 829  Fitzpatrick scale 1-2 | 449 (54.2 %) | 44 (47.8 %) | 405 (55.0 %) | 0.20 |
| Season at 18 months questionnaire, *n*=881  November to April | 383 (43.5 %) | 75 (52.1 %) | 308 (41.8 %) | **0.02** |
| Exclusive breastfeeding, weeks, *n* = 874 | 14 [15] | 12 [16] | 14 [14] | 0.08 |
| Vitamin D suppl. at age 5 years†, *n* = 762  ≤ 1 time per week, *n* (%)  ≥ 2 times per week, *n* (%) | 378 (49 .6 %)  384 (50.4 %) | 56 (54.4 %)  47 (45.6 %) | 322 (48.9 %)  337 (51.1 %) | 0.30 |
| s-25(OH)D 5 years, nmol/l | 72.0 [32.4] | 67.7 [38.5] | 72.8 [31.2] | 0.31 |
| Holiday weeks‡, *n* = 881 | 0 [0] | 0 [0] | 0 [0] | **0.001** |
| Physical activity, *n* = 791  Less active, *n* (%)  As active, *n* (%)  More active, *n* (%) | 64 (8.1 %)  533 (67.4 %)  194 (24.5 %) | 5 (9.3 %)  31 (57.4 %)  18 (33.3 %) | 59 (8.0 %)  502 (68.1 %)  176 (23.9 %) | 0.25 |
| Meat intake, days/month, *n* = 765 | 23 [10] | 20 [7] | 23 [10] | 0.69 |
| Daily dairy consumption *n* = 781  ≥ 3 dl/day | 438 (56.1 %) | 22 (50.0 %) | 416 (56.5 %) | 0.40 |
| Vitamin D suppl. at age 7 years†, *n* = 783  ≤ 1 time per week, *n* (%)  ≥ 2 times per week, *n* (%)  Unsure of frequency, *n* (%) | 411 (52.5 %)  335 (42.8 %)  37 (4.73 %) | 23 (48.9 %)  21 (44.7 %)  3 (6.4 %) | 388 (52.7 %)  314 (42.7 %)  34 (4.6 %) | 0.80 |
| Age at DXA scan, years, *n* = 881 | 7.08 [0.08] | 7.09 [0.08] | 7.07 [0.08] | **0.002** |
| TBLH BMD, g/cm^2^, *n* = 881 | 0.613 (0.049) | 0.615 (0.049) | 0.613 (0.049) | 0.61 |
| TBLH BMC, g, *n* = 881 | 621.400 [134.750] | 615.136 [122.305] | 622.237 [135.702] | 0.69 |
| TBLH BA, cm^2^, *n* = 881 | 1028.37 (93.801) | 1032.57 (93.866) | 1027.55 (93.830) | 0.56 |
| TBLH *z*-score, *n* = 881 | 0.364 (0.816) | 0.363 (0.828) | 0.364 (0.305) | 0.99 |
| Height, cm, *n* = 881 | 125.6 (5.2) | 125.7 (5.1) | 125.6 (5.2) | 0.83 |
| Weight, kg, *n* = 881 | 24.1 [4.7] | 24.4 [4.6] | 24.0 [4.7] | 0.37 |
| Child BMI, kg/m^2^, *n* = 881 | 15.4 [1.9] | 15.5 [2.1] | 15.3 [1.8] | 0.30 |

Abbreviations: suppl., supplementation; s-25(OH)D, serum 25-hydroxyvitamin D; DXA, dual-energy X-ray absorptiometry; TBLH BMD, total-body-less-head bone mineral density; TBLH BMC, total-body-less-head bone mineral content; TBLH BA, total-body-less-head bone area;

*Mothers carrying their first child during the studied pregnancy.

†Data on vitamin D supplementation in pregnancy and at ages 5 and 7 years could not be specified in more details with regards to doses.

‡Weeks during the winter half-year spend by the child (age 3–7 years) in countries with average monthly UV index high enough that the skin produces vitamin D.

Differences between those with complete covariate data and those with incomplete data were tested using two-sample Wilcoxon rank sum (Mann-Whitney) test on non-normally distributed variables, Pearson χ^2^ test on categorical variables, and two-sample *t-*test with equal variances on normally distributed variables.

| **Supplementary Table 5:** Participant vs. non-participant analyses. | | | | | |
| --- | --- | --- | --- | --- | --- |
| **Variable** | **All**  [IQR]  *n =* 2,640 | **Participants**  [IQR]  *n =* 1,194 | **Non-participants** [IQR]  *n =* 1,446 | ***p-*value** | |
| Age of mother, years | 30 [6], *n* = 2,526  2,556  805 (31.5 %)  1,243 (48.6 %)  508 (19.9 %) | 30 [6], *n* = 1,194 | 30 [6], *n* = 1,332 | | **0.001** |
| Maternal educational level, total *n*  Low, *n* (%)  Intermediate, *n* (%)  High, *n* (%) |  | 1,181  322 (27.3 %)  591 (50.0 %)  268 (22.7 %) | 1,375  483 (35.1 %)  652 (47.4 %)  240 (17.4 %) | | **0.001** |
| Parity, total *n*  Unipara*, *n* (%) | 2,516  1,392 (55.3 %) | 997  620 (51.93 %) | 1,065  772 (58.4 %) | | **0.001** |
| Smoking during pregnancy, total *n*  Yes, *n* (%) | 2,502  124 (5.0 %) | 1,187  50 (4.2 %) | 1,315  74 (5.63 %) | | 0.10 |
| Alcohol during pregnancy, total *n*  Yes, *n* (%) | 1,538  167 (10.9 %) | 781  93 (11.9%) | 757  74 (9.8 %) | | 0.18 |
| Vitamin D suppl. (preg)†, total *n*  Yes, *n* (%) | 1,213  1,054 (86.9 %) | 607  520 (85.7 %) | 606  534 (88.12 %) | | 0.20 |
| s-25(OH)D early preg, nmol/l | 65.8 [29.0], *n =* 1,219 | 64.7 [28.4], *n =* 600 | 67.0 [29.8], *n =* 619 | | **0.01** |
| s-25(OH)D late preg, nmol/l | 78.7 [37.0], *n =* 1,349 | 78.8 [35.9], *n =* 679 | 78.6 [38.5], *n =* 670 | | 0.80 |
| s-25(OH)D cord, nmol/l | 45.4 [29.9], *n =* 2,063 | 46.0 [29.7], *n =* 997 | 44.4 [29.8], *n =* 1,066 | | 0.23 |
| Sex, total *n*  Males, *n* (%) | 2,533  1,344 (53.1 %) | 1,194  624 (52.3 %) | 1,339  720 (53.8 %) | | 0.45 |
| Gestation age at birth, weeks | 40.1 [1.9], *n* = 2,520 | 40.1 [1.9], *n* = 1,194 | 40.1 [1.9], *n* = 1,326 | | 0.77 |
| Body weight at birth, g | 3,545 [607], *n* = 2,515 | 3,550 [680], *n* = 1,193 | 3,530 [660], *n* = 1,322 | | 0.14 |
| Body length at birth, cm | 52.0 [3.0], *n* = 2495 | 52.0 [3.0], *n* = 1184 | 52.0 [3.0], *n* = 1311 | | **0.02** |
| Skin tone, Fitzpatrick scale, total *n*  Fitzpatrick scale 1–2, *n* (%) | 1847  962 (52.1 %) | 1,114  599 (53.8 %) | 733  363 (49.5 %) | | 0.07 |
| Exclusive breastfeeding, weeks | 12 [16], *n* = 1,989 | 12 [16], *n* = 1,048 | 11 [15], *n* = 941 | | **0.001** |
| Season, 18 mo questionnaire, total, *n*  November to April, *n* | 1613  710 (44.0 %) | 908  398 (43.8 %) | 705  312 (44.3 %) | | 0.87 |
| Adherence, vitamin D suppl. (18 mo), *n*  High adherence‡, *n* (%) | 1574  816 (51.8 %) | 881  475 (53.9 %) | 693  341 (49.2 %) | | 0.06 |
| Physical activity level, total *n*  Low, *n* (%)  Intermediate, *n* (%)  High, *n* (%) | 1,434  102 (7.1 %)  967 (67.4 %)  365 (25.5 %) | 1,030  76 (7.4 %)  694 (67.4 %)  260 (25.2 %) | 404  26 (6.4 %)  273 (67.6 %)  105 (26.0 %) | | 0.81 |
| Daily dairy consumption, total *n*  ≤ 3 dl/day, *n* (%) | 1,413  627 (44.4 %) | 1,017  442 (43.5 %) | 396  185 (46.7 %) | | 0.27 |
| Vitamin D suppl. (5 years)†, total *n*  ≥ 2 times per week, *n* (%) | 1,571  776 (49.4 %) | 960  481 (50.1 %) | 611  295 (48.3 %) | | 0.48 |
| s-25(OH)D 5 years, nmol/l | 71. [32.4], *n* = 931 | 71.9 [32.8], *n* = 658 | 72.0 [30.5], *n* = 273 | | 0.82 |
| Holiday weeks§ | 0 [0], *n* = 2,241 | 0 [0], *n* = 1,193 | 0 [0], *n* = 1,048 | | **0.001** |
| Vitamin D suppl. (7 years)†, total *n*  ≥ 2 times per week, *n* (%) | 1,417  614 (43.3 %) | 1,021  442 (43.3 %) | 396  172 (43.4 %) | | 0.81 |
| Abbreviations: vs., versus; suppl., supplementation; preg, pregnancy; s-25(OH)D, serum 25-hydroxyvitamin D; mo, months.  *Mothers carrying their first child during the studied pregnancy.  †Data on vitamin D supplementation in pregnancy and at ages 5 and 7 years could not be specified in more details with regard to doses.  ‡ High adherence was defined as consuming 10 µg vitamin D supplementation 6–7 times per week during at least 80 % of the observation time, and non-adherent as otherwise.  §Weeks during the winter half year spend by the child (age 3–7 years) spend in countries with average monthly UV index high enough that the skin produces vitamin D.  Differences between the participants and the non-participants groups were tested using two-sample Wilcoxon rank sum (Mann-Whitney) test on non-normally distributed variables, and Pearson χ^2^ test on categorical variables. Inter-quartile ranges are given for means in square brackets [IQR]. | | | | | |
